# Insulin sensitivity is normalized in the third generation (F3) offspring of developmentally programmed insulin resistant (F2) rats fed an energy-restricted diet

**DOI:** 10.1186/1743-7075-5-26

**Published:** 2008-10-17

**Authors:** Daniel C Benyshek, Carol S Johnston, John F Martin, William D Ross

**Affiliations:** 1 Department of Anthropology, University of Nevada Las Vegas, 4505 Maryland, Parkway, Box 455003, Las Vegas, NV 89154-5003, USA; 2 Department of Nutrition, Arizona State University, Mesa, AZ, USA; 3 School of Human Evolution and Social Change, Arizona State University, Tempe, AZ, USA; 4 College of Medicine, University of Arizona, Tucson, AZ, USA

## Abstract

**Background/Aims:**

The offspring and grandoffspring of female rats fed low protein diets during pregnancy and lactation, but fed nutritionally adequate diets thereafter, have been shown to exhibit altered insulin sensitivity in adulthood. The current study investigates the insulin sensitivity of the offspring and grandoffspring of female rats fed low protein diets during pregnancy, and then maintained on energy-restricted diets post weaning over three generations.

**Methods:**

Female Sprague Dawley rats (F0) were mated with control males and protein malnourished during pregnancy/lactation. F1 offspring were then weaned to adequate but energy-restricted diets into adulthood. F1 dams were fed energy-restricted diets throughout pregnancy/lactation. F2 offspring were also fed energy-restricted diets post weaning. F2 pregnant dams were maintained as described above. Their F3 offspring were split into two groups; one was maintained on the energy-restricted diet, the other was maintained on an adequate diet consumed *ad libitum *post weaning.

**Results:**

F2 animals fed energy-restricted diets were insulin resistant (p < 0.05), while the insulin sensitivity of their F3 offspring equaled and surpassed that of controls on both the energy-restricted and adequate *ad libitum *postweaning diets (p < 0.05).

**Conclusion:**

Maternal energy-restriction did not consistently program reduced insulin sensitivity in offspring over three consecutive generations. The reasons for this remain unclear. It is possible that the intergenerational transmission of developmentally programmed insulin resistance is determined in part by the relative insulin sensitivity of the mother during pregnancy/lactation.

## Background

An increasing number of experimental animal studies have demonstrated the intergenerational effects of developmental programming on a variety of phenotypic traits, including birth weight, blood pressure, and glucose metabolism. While the investigation of the multigenerational effects of developmentally programmed traits is widening, very few studies have explored the potential for these traits to be transmitted beyond the second (F2) generation, or with post weaning diets other than adequate-control [1]. A recent study reported that glucose metabolism is altered in the adequately-nourished offspring (F1 through F3 generation) of dams (F0) malnourished during gestation and lactation [2]. Here we extend those findings with a pilot study examining the intergenerational effects of energy-restricted diets on developmentally programmed insulin sensitivity. As in previous studies, we found that the adult offspring (F1) of normal dams (F0) fed a low protein diet while pregnant and nursing were low birth weight and insulin sensitive, but that their adult offspring (F2) were less insulin sensitive than control animals. These animals (F1 and F2 generations) were maintained on a nutritionally adequate (i.e., standard 'control' chow) but energy-restricted diet (70% of control *ad libitum *consumption) throughout life. However, the F3 generation, showed normal – and even *enhanced *insulin sensitivity – regardless of whether they were fed the same nutritionally-adequate but energy-restricted diet, or given *ad libitum *access to the nutritionally adequate diet post weaning.

## Materials and methods

Sprague Dawley rats consumed either a nutritionally adequate diet (20% protein; TD 91352, Harlan Teklad, Madison, WI), or an isocaloric, low protein diet (8% protein; TD 93033, Harlan Teklad, Madison, WI) from day one of pregnancy through lactation. Litters were culled to 8 pups/litter after birth weights were recorded. Pups whose mothers were protein malnourished *in utero *consumed an adequate but energy-restricted (70% of *ad libitum *control daily consumption) diet postweaning. The dams of control pups were adequately nourished throughout pregnancy and their offspring consumed an adequate diet *ad libitum *postweaning. (An F2 generation of control animals was also bred, with no statistically significant differences observed between F1 and F2 control animals with respect to any body weight or metabolic measures.) At ~75 days of age control and experimental animals were food deprived overnight and fasting glucose and insulin levels were measured. Animals were sacrificed under CO2 anesthesia. Blood was collected by cardiac puncture. Four female median body-weight first-generation (F1) rats whose mothers were protein malnourished during pregnancy and while nursing were selected from the experimental group at ~75 days of age, mated with control breeder males, and maintained on the energy-restricted diet throughout gestation and lactation. Their offspring, the F2 generation, also consumed a nutritionally adequate but energy-restricted diet post-weaning. At ~75 days of age fasting glucose and insulin values were obtained from the F2 rats as described. A final generation of animals (F3) was bred from control breeder males and four median body-weight F2 dams whose grandmothers (F0) had been protein malnourished throughout pregnancy and lactation. F3 animals were then split into two groups. One group was maintained on the energy-restricted diet, while the other was given *ad libitum *access to the adequate diet post weaning. At ~75 days of age, all F3 animals were tested as described (Table 1). This research was approval by the Institutional Animal Care and Use Committee (IACUC) at Arizona State University and the University of Nevada Las Vegas. Animals were maintained in accordance with the 'Guide for the Care and Use of Laboratory Animals' of the National Research Council (7^th ^ed., 1996).

**Table 1:** Research Design

**Generation**	**Breeding Dam Prenatal/Nursing Diet**	**Offspring Post weaning Diet**
F0	Low Protein	Energy-Restricted

F1	Energy-Restricted	Energy-Restricted

F2	Energy-Restricted	Energy-Restricted

F3	Energy-Restricted	F3.1 Energy-Restricted
		
		F3.2 Nutritionally-Adequate

Controls	Nutritionally-Adequate	Nutritionally-Adequate

Nutritionally Adequate diet – *ad libitum *(20% protein; TD 91352, Harlan Teklad, Madison, WI); Low Protein diet – *ad libitum *(isocaloric, 8% protein; TD 93033, Harlan Teklad, Madison, WI); Energy-Restricted – 70% control animal consumption of Nutritionally Adequate diet.

## Results

**Figure 1:**
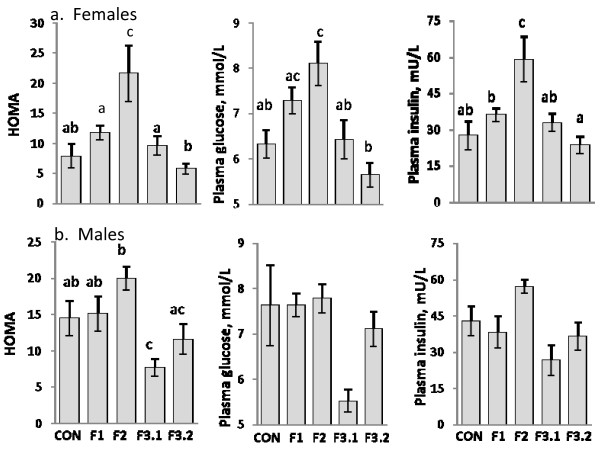
**HOMA, Fasting Glucose, and Fasting Insulin**. HOMA, fasting glucose, and fasting insulin for female (a.) and male (b.) control rats (CON); rats whose dams consumed low-protein diets while pregnant and nursing (F1), offspring of F1 rats (F2); and offspring of F2 rats (F3.1 and F3.2). Control rat dams consumed adequate diets *ad libitum *during pregnancy and while nursing, and control offspring consumed adequate diets *ad libitum *postweaning. All F1, F2 and F3 offspring were fed nutritionally adequate but energy-restricted diets (70% of control *ad libitum *consumption) postweaning with the exception of the F3.2 offspring that were fed the control diet *ad libitum*. Bars with different letters differ significantly (p < 0.05, significant univariate ANOVA with LSD post-hoc analysis).

As in previous reports [1,3], the mean birth weight of both male and female F1 animals in our study were significantly lower (p < 0.05) than control animals (Table 2). Mean birth weights of F2 and F3 male and female animals did not differ significantly from control animals, although the mean weaning and 75 day weights were both significantly lower than those of controls animals, respectively. Our results (Figure 1) also show that the effects of maternal protein malnutrition during prenatal and suckling periods on glucose homeostasis of F1 and F2 offspring are consistent with previous studies where offspring were weaned onto adequate (control) diets: normal insulin sensitivity in F1 animals [4,5], and hyperinsulinemia/insulin resistance in F2 animals [2,3]. Unexpectedly, our results also showed that the adult F3 offspring of insulin resistant F2 dams fed an energy-restricted diet during pregnancy and lactation and maintained on the restricted diet post weaning (F3.1), were equally insulin sensitive (females), or *more *insulin sensitive (males), than control animals, as measured by Homeostatic model assessment (HOMA). The insulin sensitivity of male and female experimental F3 offspring given *ad libitum *access to adequate diets post weaning (F3.2) improved to that of controls animals. While the general trends of intergenerational changes in glucose-metabolism are similar for both F1 – F3 males and females, the differences are more pronounced for females. Among F2 females, mean HOMA, insulin and glucose levels are all significantly (p < 0.05) higher than control values, with HOMA and insulin values over 250 and 200 percent, respectively, compared to controls. Among both post weaning diet groups of adult F3 females (F3.1, F3.2), however, HOMA, glucose and insulin levels all effectively return to values of adequately fed control animals. Insulin sensitivity, as measured by HOMA, also improved dramatically among F3 males fed an energy-restricted diet post weaning (F3.1) relative to F2 males, and actually exceeds the insulin sensitivity of control animals (p < 0.05). Insulin sensitivity of experimental F3 males given *ad libitum *access to an adequate diet post weaning (F3.2) also improved relative to F2 males, but improves only to that of control animals.

**Table 2:** Birth Weight, Weaning Weight, and Adult Weight

**Males**	**Birth Weight (g)**	**Weaning Weight (g)**	**75 d Weight (g)**
Controls (10)	7.1 ± 0.4 a	68.7 ± 1.4 a	449.0 ± 8.8 a

F1 (7)	5.1 ± 0.1 b	23.9 ± 1.7 b	257.4 ± 11.5 b

F2 (7)	6.6 ± 0.2 ac	29.0 ± 0.8 c	276.4 ± 16.2 b

F3.1 (7)	5.8 ± 0.2 bc	29.1 ± 0.9 c	250.0 ± 6.5 b

F3.2 (7)	6.6 ± 0.3 ac	31.9 ± 0.8 c	337.0 ± 9.4 c

**Females**			

Controls (3)	7.7 ± 0.9 a	60.0 ± 1.7 a	261.3 ± 3.2 a

F1 (7)	5.3 ± 0.3 b	26.3 ± 1.0 bc	173.7 ± 7.1 b

F2 (3)	7.0 ± 0.0 ac	29.3 ± 0.3 c	187.7 ± 8.8 b

F3.1 (6)	6.0 ± 0.3 bc	26.0 ± 0.9 b	182.8 ± 5.5 b

F3.2 (5)	6.4 ±0.2 c	28.8 ± 0.7 c	223.6 ± 2.3 c

Mean birth weights, 14 d weaning weights, and 75 d weights for control rats and rats whose dams consumed low-protein diets while pregnant and nursing (F1), offspring of F1 rats (F2); and, offspring of F2 rats (F3.1 and F3.2). Control rat dams consumed adequate diets *ad libitum *during pregnancy and while nursing, and control offspring consumed adequate diets *ad libitum *postweaning. All F1, F2 and F3 offspring were fed nutritionally adequate but energy-restricted diets (70% of control *ad libitum *consumption) postweaning with the exception of the F3.2 offspring that were fed the control diet *ad libitum*. (Sample size shown in parentheses.) Means (± SE) in columns with different letters differ significantly (p < 0.05, significant univariate ANOVA with LSD post-hoc analysis).

## Discussion

The results from previous animal studies have shown that nutritional insults during pregnancy and lactation are linked to glucose intolerance via either hyperinsulinemia/insulin resistance, *or *decreased insulin secretory capacity, depending on the type and timing of the prenatal nutritional insult. Maternal low-protein diets during pregnancy followed by control diets post weaning [2,5], in addition to energy restricted prenatal diets during the latter half of pregnancy followed by control diets post weaning [6,7] are associated with insulin-sensitive offspring, but with reduced insulin secretory capacity. The findings reported here for F1 animals are consistent with the maternal protein malnutrition animal model that are linked with impaired glucose tolerance among the mature offspring – but without accompanying hyperinsulinemia/insulin resistance. In contrast, other previous single-generation animal studies have shown that maternal energy-restricted diets fed *throughout *pregnancy lead to hyperinsulinemia/insulin resistance in adult offspring when followed by adequate and hypercaloric diets post weaning [8,9]. Similarly, we report here that reduced insulin sensitivity and hyperinsulinemia are also evident in (F2) animals whose mothers were fed energy-restricted diets throughout pregnancy/lactation and are then maintained on the same energy restricted diet post weaning. Why insulin sensitivity normalizes in the F3 *offspring *of such developmentally-programmed insulin resistant F2 females, however, even though they were exposed to the same prenatal and post weaning dietary protocols as their mothers (energy-restricted/energy-restricted) remains to be determined. This is especially puzzling given that insulin resistance is generally characterized as an 'adaptive' phenotypic trait in nutrient poor environments [10]. Based on the results presented here, it is possible that the insulin sensitivity of the *mother *fed an energy-restricted diet during pregnancy is a critical variable (i.e., offspring of insulin-resistant mothers fed energy-restricted diets develop normal insulin sensitivity, while the offspring of insulin-sensitive mothers fed energy-restricted diets during pregnancy become insulin resistant). In the current study, the reason for this may be that the maternal insulin resistance of pregnant dams fed an energy-restricted diet increases the availability of nutrients for the fetus – in effect normalizing fetal nutrition and development – while the relative insulin sensitivity of pregnant dams fed an energy-restricted diet makes fewer nutrients available for the developing fetus and programs for insulin resistance in the offspring. For F3 animals whose (F2) mothers were fed energy-restricted diets during pregnancy/lactation, but that were fed adequate diets *ad libitum *post weaning (F3.2), their improved insulin sensitivity, despite significant (p < 0.05) increases in adult body weight, suggests that maternal diet during gestation/lactation, and not post weaning diet or body weight is the critical variable determining relative insulin sensitivity/resistance. This is of particular interest, given that our group has previously shown that the adequately-nourished grand-offspring (F3) of (F1) rats malnourished during gestation and perinatal life remain significantly less insulin sensitive than control animals [2], suggesting that it is the energy-restriction during pregnancy and lactation in the current study that results in the improved insulin sensitivity of F3 animals. Sex differences are evident in the data in that changes in glucose-insulin metabolism were more pronounced, and more often reached the level of statistical significance, among females than among males. The overall pattern of intergenerational change in glucose-metabolism is similar for both F1 – F3 males and females, however, and among both sexes, insulin resistance (as measured by HOMA), along with fasting insulin and glucose levels, peak in the F2 generation. This leaves the extent of the reduction of insulin resistance between F3.1 (energy restricted post weaning diet) and F3.2 (*ad libitum *post weaning diet) male and female animals as the only other significant sex-based difference. Here, among both males and females, insulin sensitivity improves dramatically among all F3 animals (whose mothers were fed an energy restricted diet during pregnancy), but among females the larger improvement is among the F3.2 (*ad libitum*) post weaning diet; whereas among males, insulin sensitivity is most improved among animals fed the F2.1 (energy-restricted post weaning) diet. These sex-based differences should be interpreted with caution, however, because while differences exist in the degree of improved insulin sensitivity between F3 males and females by post weaning diet, among F3.1 and F3.2 females HOMA values do not differ significantly from female controls; and HOMA values of F3.1 and F3.2 males do not differ significantly from each other.

## Conclusion

Contrary to findings in previous studies in which the offspring and grandoffspring of female rats fed low protein diets during pregnancy and lactation, but fed nutritionally adequate diets thereafter, exhibit altered insulin sensitivity in adulthood [2], the current study found that maternal energy-restricted diets during pregnancy and nursing, followed by post weaning energy restricted diets, did not consistently program reduced insulin sensitivity among F2 and F3 offspring of maternally protein malnourished dams (F1). Instead, F2 animals were hyperinsulinemic/insulin resistant, while F3 animals subjected to the identical diets throughout their life course were at least as insulin sensitive as control animals. The reasons for this remain unclear. It is possible that the intergenerational transmission of developmentally programmed insulin resistance is determined in part by the relative insulin sensitivity of the mother during pregnancy/lactation. Further research is needed on the metabolic effects of pre and postnatal diets, varying in nutrient composition and over multiple generations, in order to better understand the fundamental principles of the transgenerational transmission of developmentally programmed traits. Such further investigation of the variability in the intergenerational transmission of developmentally programmed traits is clearly warranted, not only as a matter of etiological significance, but for its relevance to possible future disease prevention applications among humans.

## Competing interests

The authors declare that they have no competing interests.

## Authors' contributions

DCB designed the study, collected assay blood samples, and drafted the manuscript. CSJ collected assay blood samples, and performed the statistical analysis and helped draft the manuscript. JFM contributed to study design and data analysis. WDR helped with the study design, collected assay blood samples, and was responsible for daily animal husbandry. All authors read and approved the final manuscript.
